# The key to life satisfaction in emerging adults: unlocking the secrets of self-efficacy and physical activity

**DOI:** 10.3389/fpubh.2024.1431255

**Published:** 2024-11-13

**Authors:** Lin Luo

**Affiliations:** School of Physical Education, Guizhou Normal University, Guiyang, China

**Keywords:** emerging adulthood, life satisfaction, self-efficacy, physical activity, college students

## Abstract

**Purpose:**

This study aimed to explore the relationships among characteristics of emerging adulthood, self-efficacy, and physical activity behaviors, and how these factors collectively influence the life satisfaction of college students. By delving into the interconnections between these elements, insights for relevant interventions and policy formulation can be provided.

**Methods:**

In this cross-sectional study conducted from November to December 2022, a total of 3,387 Chinese college students were surveyed via the Maike electronic questionnaire platform. The Inventory of the Dimensions of Emerging Adulthood (IDEA), General Self-Efficacy Scale (GSE-8), Physical Activity Rating Scale (PARS-3), and a single-item life satisfaction question were utilized for assessments. Data analysis was performed using the Bollen–Stine bootstrap method, with the aid of Amos software (version 26.0) and IBM SPSS (version 26.0).

**Results:**

Instability, possibility, and self-exploration were significantly associated with self-efficacy, which in turn influenced life satisfaction. Factors such as possibility and instability were related to physical activity behaviors, further predicting life satisfaction. Characteristics of emerging adulthood indirectly affected life satisfaction through self-efficacy and physical activity behaviors.

**Conclusion:**

Characteristics of emerging adulthood enhance life satisfaction by bolstering self-efficacy and promoting physical activity behaviors.

## Introduction

University life represents a critical developmental stage for emerging adults, profoundly influencing their overall life satisfaction (1). During this period, students encounter various challenges related to academic performance, interpersonal relationships, and career development while simultaneously beginning to explore their identities and values (1). Although numerous studies have examined the factors influencing life satisfaction, including personal traits and social support (2–6), the relationship between core developmental characteristics of emerging adulthood (such as autonomy and responsibility) and life satisfaction remains insufficiently elucidated (7, 8). Recent research indicates a positive correlation between self-exploration and responsibility with the life satisfaction of emerging adults in China, while emotional instability shows a negative correlation (9–11).

Self-efficacy and physical activity play significant roles in the life satisfaction of university students. Self-efficacy refers to an individual’s confidence in their ability to accomplish specific tasks (12). This belief is vital for setting goals and managing challenges in university (13). Students with high self-efficacy are more resolute in pursuing their goals and strongly believe they can effectively handle challenges related to academics, interpersonal relationships, and future careers (14). This confidence enables them to overcome adversity and achieve success, positively impacting their life satisfaction (12). Furthermore, studies have also shown a close relationship between physical activity and life satisfaction among university students (15, 16). Regular participation in physical activity not only improves physical health and reduces stress but also enhances self-efficacy, further affecting life satisfaction (8). Physical activity provides students with opportunities to relieve stress, foster positive emotions, and promote healthy interpersonal relationships, which together further enhance life satisfaction (15, 16).

Emerging adulthood is typically defined as the transitional stage between the ages of 18 and 29, marking the shift from adolescence to adulthood (11). During this phase, university students actively explore lifestyle and career paths, necessitating strong self-efficacy and physical activity to cope with uncertainties and challenges in life (17–20). Research has found that identity exploration, particularly in emotional and vocational domains, positively correlates with the life satisfaction of emerging adults (21, 22). This finding suggests that a deeper understanding and exploration of oneself, goals, and passions can enhance feelings of achievement and fulfillment in life (21). However, emotional and vocational instability experienced by emerging adults can negatively affect their life satisfaction (10, 11). This unpredictability may lead to challenges and stress, thereby impacting overall well-being (22).

Belief in potential pathways and opportunities for the future is associated with higher life satisfaction, and maintaining an optimistic attitude contributes to enhanced overall well-being (23). Additionally, the awareness of responsibility, which refers to an individual’s cognition regarding their behaviors and obligations, significantly interacts with life satisfaction. Research has indicated that a high sense of responsibility can elevate an individual’s life satisfaction, and vice versa (23, 24). Notably, the relationship between developmental characteristics of emerging adulthood and life satisfaction may be moderated by factors such as social support, academic achievement, and career planning (25, 26), providing important insights into supporting the well-being of university students and enhancing life satisfaction.

The interplay between characteristics of emerging adulthood, self-efficacy, and physical activity is complex; these factors interact to collectively influence the life satisfaction of university students (12, 23–28). By exploring the connections among these factors, a deeper understanding of university students’ life satisfaction can be achieved, offering valuable guidance for relevant interventions and policy developments. Thus, comprehending how these factors interact will provide guidance for formulating strategies aimed at enhancing the well-being of students during this critical developmental stage.

**Figure 1:**
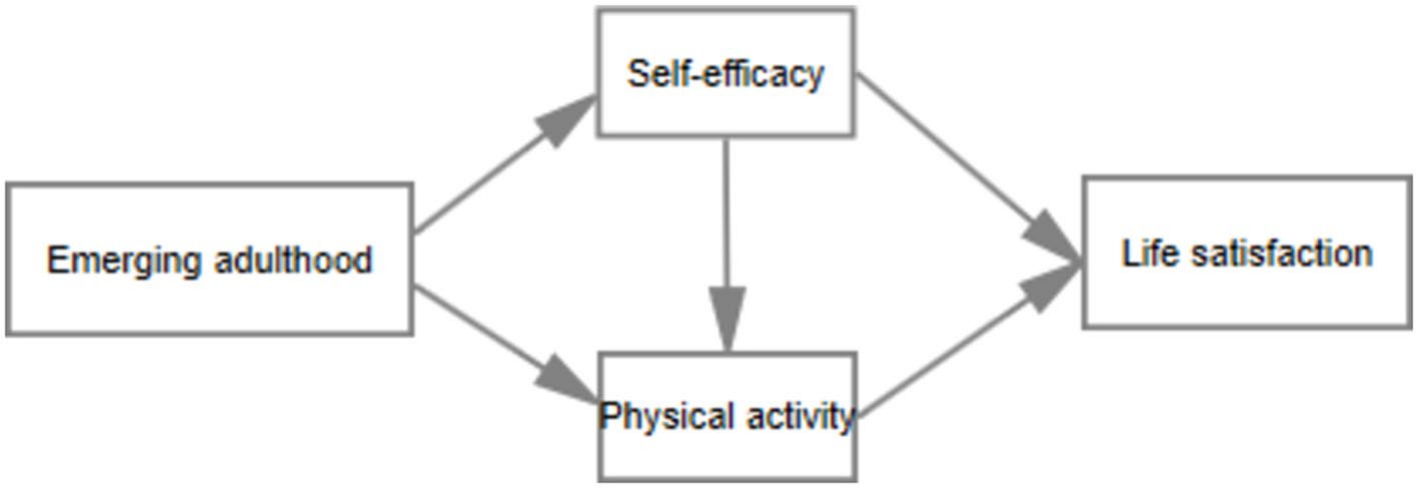
Hypothesized model.

Based on theories of developmental psychology, mental health, social support, and self-determination, the following hypotheses are proposed: the developmental characteristics of emerging adults, particularly in terms of autonomy, responsibility, maturity, and self-exploration, may indirectly influence their life satisfaction by enhancing self-efficacy and physical activity (Figure 1). Three potential pathways are suggested: (i) emerging adult characteristics → self-efficacy → life satisfaction; (ii) emerging adult characteristics → physical activity → life satisfaction; (iii) emerging adult characteristics → self-efficacy → physical activity → life satisfaction.

## Methods

This research project (code GZNUPEI 20220524) has been approved by the Academic Ethics Committee of Guizhou Normal University. For this cross-sectional study, data was collected from November to December 2022 through the Maike electronic questionnaire platform, which is similar to REDCap. The primary author contacted collaborators working at universities in China and asked them to share the scan code of the survey with their students through WeChat, an online social platform. After obtaining informed consent, university students who were willing to participate in the study completed the survey questionnaire. All questionnaires were self-reported. These university students mainly came from several public universities in the southwest and east regions of China.

### Measures

Participants were asked to provide demographic information and respond to the main research measures. Specifically, the demographic questions included age, gender (female vs. male), ethnicity (Han vs. non-Han), and household registration (urban vs. rural). This study focused on the characteristics of emerging adults, self-efficacy, physical activity, and life satisfaction, which were measured using the IDEA-C, GSE-8, PARS-3 questionnaires, and a single-item question, respectively. These measurement tools have exhibited strong validation within the Chinese university student population, thereby affirming their appropriateness for application in this study.

### Emerging adulthood

To assess the characteristics of emerging adulthood, this study utilized the Chinese version of the Inventory of the Dimensions of Emerging Adulthood (IDEA) (11). The scale consists of four factor structures: self-exploration, instability, possibility, and responsibility, with a total of 20 items. All items were rated on a four-point Likert scale, ranging from 1 (strongly disagree) to 4 (strongly agree), with higher scores indicating higher perceived levels. The Chinese version of IDEA demonstrated good internal consistency in this study, with a Cronbach’s alpha coefficient greater than 0.77. Additionally, the test–retest reliability showed good performance, with a correlation coefficient greater than 0.49 and *p*-value less than 0.01. The scale has good reliability and validity among Chinese college students. In this study, the Cronbach’s alpha coefficient for the scale was 0.922, indicating high reliability in assessing the characteristics of emerging adulthood.

### Self-efficacy

This study used the General Self-Efficacy Scale (GSE-8) to assess participants’ levels of self-efficacy (29). The GSE-8 consists of 8 items, and all items are rated on a five-point Likert scale, ranging from 1 (strongly disagree) to 5 (strongly agree). Higher scores indicate a greater level of confidence in one’s abilities. The GSE-8 has been widely used in Chinese settings, and in this study, it demonstrated good internal consistency with a Cronbach’s alpha coefficient of 0.919. The scale has good reliability and validity among Chinese college students.

### Physical activity

This study utilized the Physical Activity Rating Scale (PARS-3), developed by Liang Deqing, to assess participants’ levels of physical activity (30). The PARS-3 includes three aspects of evaluation: intensity, duration, and frequency of participation in physical activities. The physical activity level can be calculated using the following formula: Physical Activity Level = Intensity × Duration × Frequency. In the PARS-3, intensity and frequency are rated on a scale of 1–5, corresponding to scores of 1–5, while duration is rated on a scale of 1–5, corresponding to scores of 0–4. The maximum score is 100, and the minimum score is 0. A higher score indicates a greater level of physical activity. The scale has good reliability and validity among Chinese college students.

### Life satisfaction

Although “Are you satisfied with your life?” is not a classical measurement method for life satisfaction, it is widely used and considered a simple and effective measure (31). Classical life satisfaction questionnaires typically consist of multiple items that cover different aspects and dimensions of life (32). These questionnaires assess participants’ overall sense of life satisfaction and specific evaluations of different aspects through multiple questions. However, in some survey studies and quick assessments, it is common practice to use a single question to assess overall life satisfaction (33). Therefore, in this study, the question “Are you satisfied with your life?” was used to measure life satisfaction. Participants rated their satisfaction on a five-point Likert scale, ranging from 1 (very dissatisfied) to 5 (very satisfied).

### Statistical analysis

After conducting multicollinearity tests, it was determined that all variables in the model had VIF values less than 5, indicating the absence of multicollinearity issues. Additionally, the D-W statistic was close to 2, suggesting no autocorrelation in the model, meaning there was no correlation among the sample data. The K-S test indicated that the variables related to physical activity behavior, sense of responsibility, possibility, instability, self-exploration, self-efficacy, and life satisfaction did not follow a normal distribution. Given the non-normal distribution of the variables, the maximum likelihood (ML) estimation method was not utilized, as it may lead to bias in chi-square values, fit indices, and standard errors (34), and may introduce bias in chi-square fit (35). Instead, the Bollen–Stine bootstrap method was employed to assess the overall model fit, which does not rely on maximum likelihood estimation. The assumptions of path analysis align with the requirements of structural equation modeling (SEM). Bootstrap analysis was conducted with 5,000 samples and a 95% bias-corrected confidence interval (CI). Statistical analysis was performed using Amos software (Version 26.0; Chicago: IBM SPSS).

IBM SPSS Statistics for Windows was used to compute the sample distribution, means, standard deviations, and possible correlations among variables in the theoretical model (using Pearson correlation coefficients). As suggested by Cui et al. (36), the correlation coefficients were assessed as follows: 0–0.19: no correlation, 0.20–0.39: low correlation, 0.40–0.69: moderate correlation, 0.60–0.79: high correlation, and ≥0.80: very high correlation. Additionally, Cronbach’s Alpha coefficient was used to assess the internal consistency of specific scales. According to Hair et al. (37), a Cronbach’s Alpha coefficient greater than 0.7 indicates high reliability, while coefficients between 0.6 and 0.7 are considered acceptable.

Different structural equation models were tested in the following order: (i) Emerging adulthood characteristics → self-efficacy → life satisfaction; (ii) Emerging adulthood characteristics → physical activity behavior → life satisfaction; (iii) Emerging adulthood characteristics → self-efficacy → physical activity behavior → life satisfaction. The fit of the models was determined by the following indicators (38): (i) Statistical significance level set at *α* = 0.05. (ii) Root Mean Square Error of Approximation (RMSEA): RMSEA ≤0.05 indicates good fit, ≥ 0.10 indicates poor fit. (iii) Comparative Fit Index (CFI): CFI = 1 represents the best fit, ≥ 0.95 indicates good fit, 0.90–0.95 indicates reasonable fit.

## Results

### Demographic characteristics of participants

A total of 3,387 participants voluntarily completed the survey. Among them, there were 1,129 males (33.33%) and 2,258 females (66.67%); the average age was 19.15 ± 1.81 years; 1998 participants were of Han ethnicity (58.99%), while 1,389 participants belonged to other ethnicities (41.01%); 877 participants had an urban household registration (25.89%), while 2,510 participants had a rural household registration (74.11%). The detailed results of the demographic data can be found in Table 1.

**Table 1:** Participants’ demographics and characteristics.

	Total (*n* = 3,387)	Men (*n* = 1,129)	Women (*n* = 2,258)	*p*-value
Age (years), Mean (SD)	19.15 (1.81)	19.29 (2.68)	19.09 (1.13)	0.013
Ethnicity, *n* (%)				0.824
Han	1998 (58.99%)	559 (59.26%)	1,329 (58.86%)	
None-Han	1,389 (41.01%)	460 (40.74%)	929 (41.14%)	
Residence registration, *n* (%)				0.017
City	877 (25.89%)	321 (28.43%)	556 (24.62%)	
Rural	2,510 (74.11%)	808 (71.57%)	1702 (75.38%)	

Participants’ ethnicity and residence registration are presented as *n* (%), while the average age and standard deviation are shown as Mean (SD).

### Descriptive statistics and internal reliability

Table 2 presents the means and standard deviations of the continuous variables, as well as their Cronbach’s Alpha values. The subscales related to IDEA and the GSE-8 scale demonstrate acceptable reliability (Cronbach’s Alpha coefficients = 0.70–0.95).

**Table 2:** Descriptive statistic and Cronbach’s alpha values of primary outcomes (*n* = 3,387).

	Mean	SD	Cronbach’s alpha
Self-efficacy	30.36	5.72	0.919
Physical activity	27.63	26.98	
Life satisfaction	3.57	0.91	
Responsibility	11.16	2.32	0.816
Self-exploration	24.10	3.97	0.909
Instability	14.13	2.78	0.831
Possibilities	9.02	1.69	0.786

### Correlation analysis

The correlation analysis revealed that there is a positive correlation between physical activity behavior and self-efficacy (*r* = 0.197, *p* < 0.01), as well as life satisfaction (*r* = 0.214, *p* < 0.01). Furthermore, physical activity behavior is positively correlated with a sense of responsibility (*r* = 0.079, *p* < 0.01), self-exploration (*r* = 0.073, *p* < 0.01), and possibility (*r* = 0.081, *p* < 0.01). Life satisfaction is positively correlated with self-efficacy (*r* = 0.341, *p* < 0.01), a sense of responsibility (*r* = 0.075, *p* < 0.01), self-exploration (*r* = 0.116, *p* < 0.01), and possibility (*r* = 0.081, *p* < 0.01), while negatively correlated with instability (*r* = −0.071, *p* < 0.01). Self-efficacy is positively correlated with a sense of responsibility (*r* = 0.085, *p* < 0.01), self-exploration (*r* = 0.223, *p* < 0.01), and possibility (*r* = 0.240, *p* < 0.01). The detailed results of the correlation analysis for each pair of variables can be found in Table 3.

**Table 3:** Zero-order correlations for variables (*n* = 3,387).

	1	2	3	4	5	6
1. Physical activity	1					
2. Life satisfaction	0.214	1				
3. Self-efficacy	0.197	0.341	1			
4. Responsibility	0.079	0.075	0.085	1		
5. Self-exploration	0.073	0.116	0.223	0.425	1	
6. Instability	−0.018	−0.071	−0.004	0.398	0.396	1
7. Possibilities	0.081	0.143	0.240	0.348	0.558	0.402

*p < 0.01.*

### Relationship and moderators between features of emerging adulthood and life satisfaction

**Figure 2:**
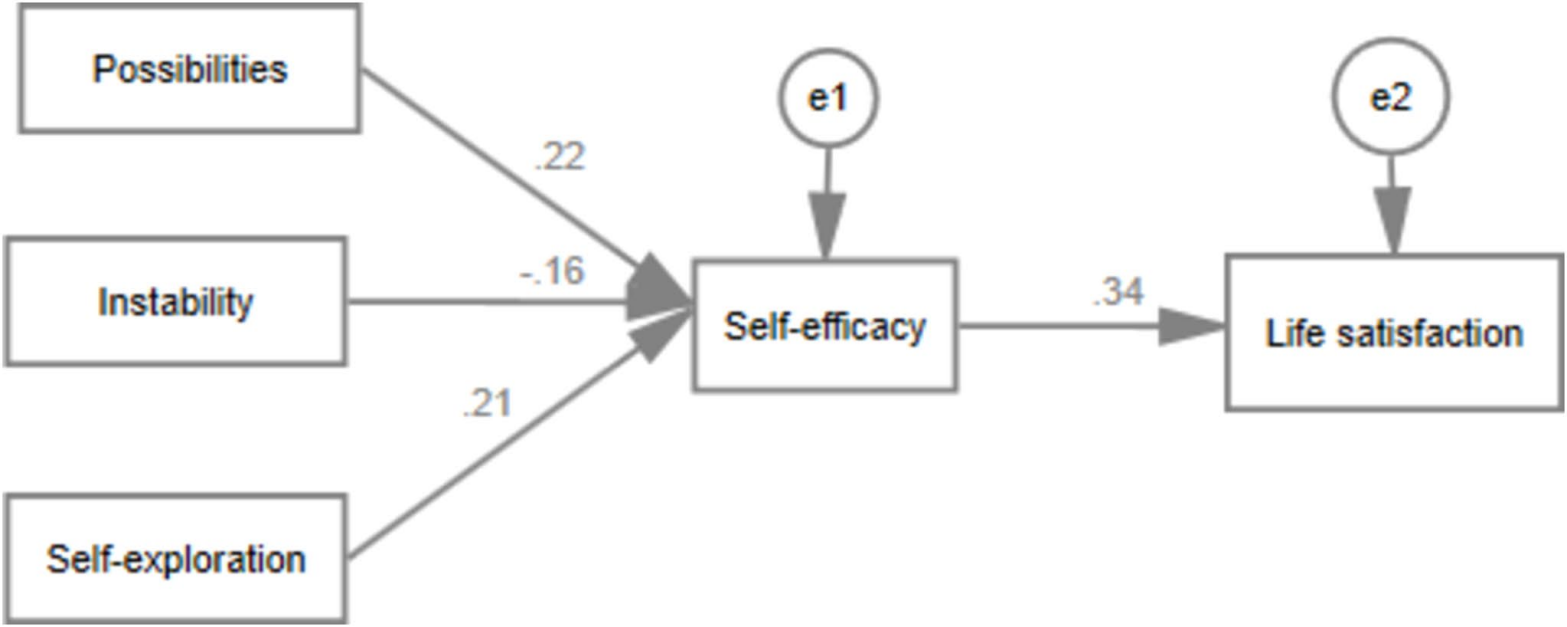
Mediating role of self-efficacy between the features of emerging adulthood and life satisfaction.

Figure 2 illustrates the significant paths in the relationship between “Features of Emerging Adulthood → Self-Efficacy → Life Satisfaction.” The research findings indicate that instability (*b* = −0.16, *p* < 0.001), possibility (*b* = 0.22, *p* < 0.001), and self-exploration (*b* = 0.21, *p* < 0.001) are statistically associated with self-efficacy. Furthermore, self-efficacy predicts the level of life satisfaction (*b* = 0.34, *p* < 0.001). The overall model fits well (*χ*^2^/df = 16.44, RMSEM = 0.068, CFI = 0.989).

**Figure 3:**
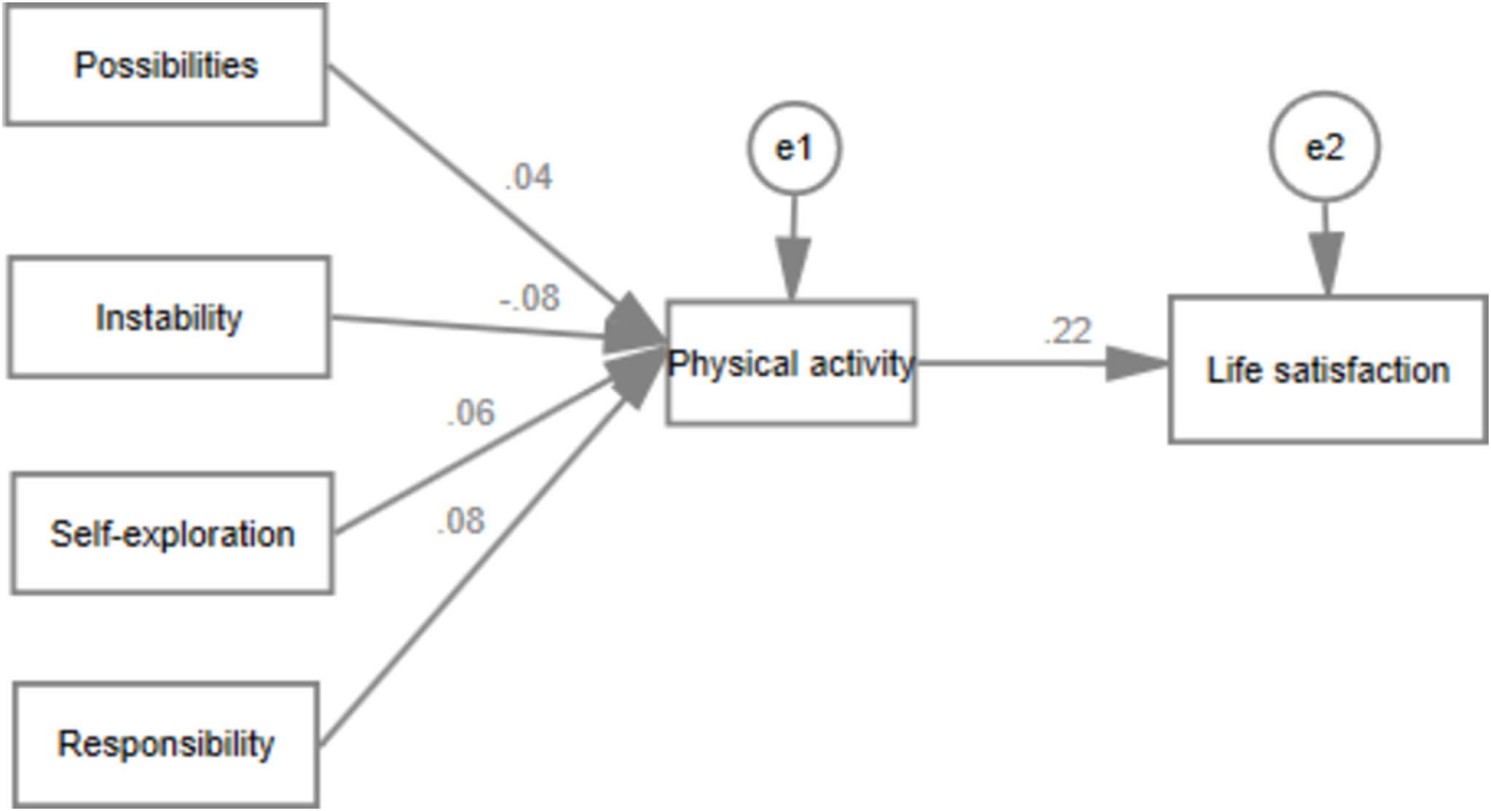
Mediating role of physical activity between the features of emerging adulthood and life satisfaction.

Figure 3 presents the significant paths in the relationship between “Features of Emerging Adulthood → Physical Activity → Life Satisfaction.” The research findings indicate that possibility (*b* = 0.04, *p* < 0.001), instability (*b* = −0.08, *p* < 0.05), self-exploration (*b* = 0.06, *p* < 0.001), and sense of responsibility (*b* = 0.08, *p* < 0.001) are statistically associated with physical activity. Furthermore, physical activity (*b* = 0.22, *p* < 0.001) predicts life satisfaction. The overall model fits well (*χ*^2^/df = 14.905, RMSEM = 0.052, CFI = 0.973).

**Figure 4:**
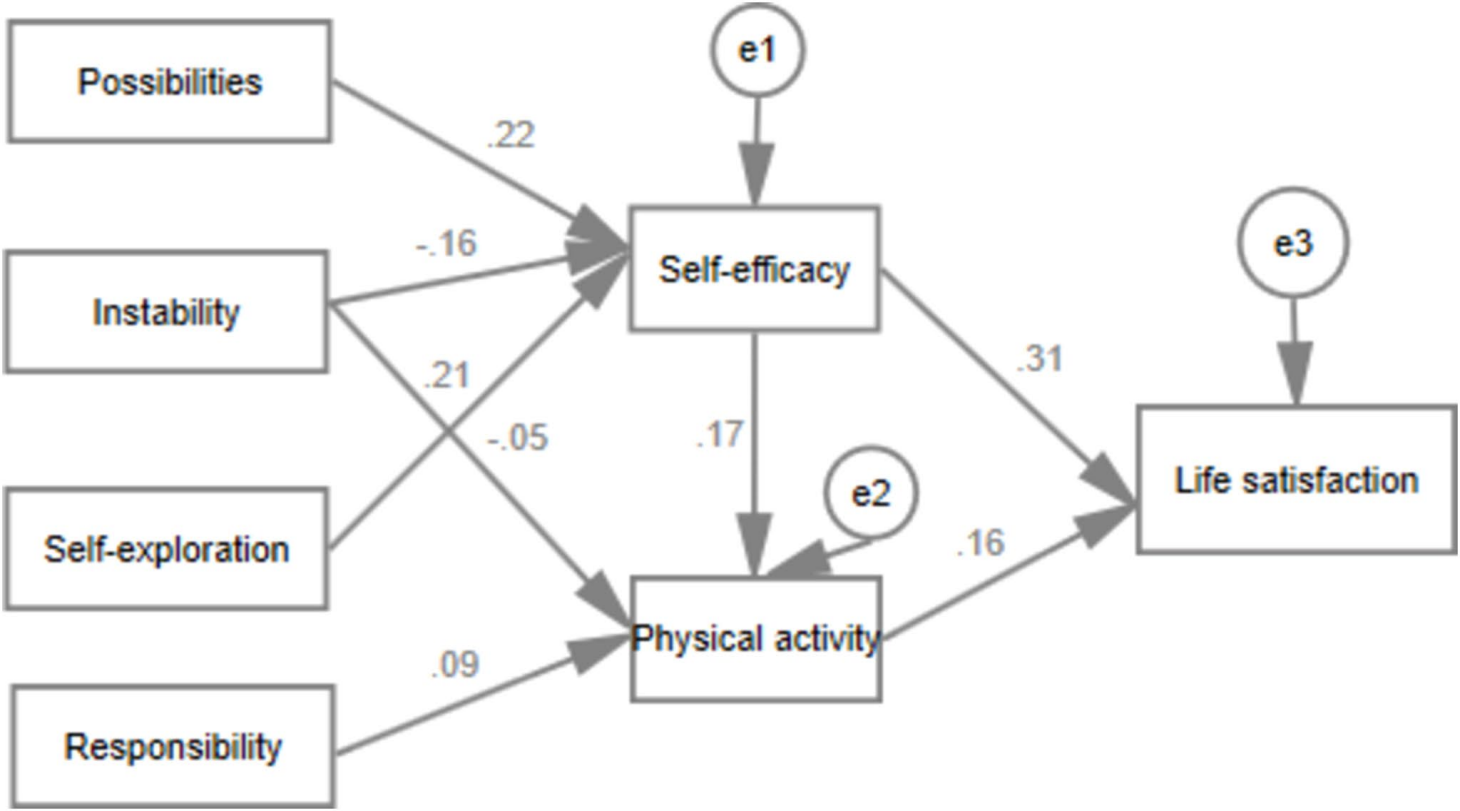
Mediating role of self-efficacy and physical activity between the features of emerging adulthood and life satisfaction.

Figure 4 illustrates the significant paths in the relationship between “Features of Emerging Adulthood → Self-Efficacy → Physical Activity → Life Satisfaction.” The research findings indicate that instability (*b* = −0.16, *p* < 0.001), possibility (*b* = 0.22, *p* < 0.001), and self-exploration (*b* = 0.21, *p* < 0.001) are statistically associated with self-efficacy. Additionally, instability (*b* = −0.05, *p* < 0.05) and sense of responsibility (*b* = 0.09, *p* < 0.001) are statistically associated with physical activity behavior. Self-efficacy predicts the level of life satisfaction (*b* = 0.31, *p* < 0.001), and physical activity behavior predicts life satisfaction (*b* = 0.16, *p* < 0.001). Self-efficacy can also predict physical activity (*b* = 0.17, *p* < 0.001). The overall model fits well (*χ*^2^/df = 8.58, RMSEM = 0.047, CFI = 0.991). The research findings validate our hypothesized model that the developmental features of emerging adulthood may indirectly influence their life satisfaction by affecting their self-efficacy and physical activity behavior.

## Discussion

This study explores how the developmental characteristics of emerging adults indirectly influence life satisfaction through self-efficacy and physical activity behavior. The three proposed mechanisms have been empirically validated.

The results indicate that the characteristics of the emerging adulthood phase, such as instability, potentiality, and self-exploration, are positively correlated with life satisfaction. This finding is consistent with existing literature, which suggests a relationship between identity exploration and higher life satisfaction during emerging adulthood (13, 17). However, this study further investigates how these characteristics influence life satisfaction through intermediary variables such as self-efficacy and physical activity behavior. Specifically, the results show a significant statistical relationship between characteristics of emerging adulthood and self-efficacy (*p* = 0.01). This outcome aligns with descriptions in the literature, indicating that growth in autonomy, responsibility, maturity, and self-exploration during emerging adulthood may positively affect self-efficacy (24).

Additionally, the significant relationship between self-efficacy and physical activity behavior (*p* = 0.01) has been confirmed, supporting previous research that indicates individuals with high self-efficacy are more likely to engage in physical activity (25, 28). By providing specific statistical data, this study deepens the understanding of the strength and direction of these relationships. Finally, a significant positive correlation between physical activity behavior and life satisfaction was revealed (*p* = 0.01), which is consistent with prior research indicating a positive relationship between regular physical exercise and higher life satisfaction (32, 33). In this context, the study further explores how other variables, such as self-efficacy, modulate this relationship.

The findings reveal that developmental characteristics, particularly emotional instability, potentiality, and self-exploration, have a clear connection with life satisfaction. These characteristics may indirectly influence life satisfaction by enhancing self-efficacy and promoting physical activity behavior. During this critical developmental stage, emerging adults experience identity exploration and construction, which may enhance their self-efficacy and motivate participation in physical activities. Previous studies have shown that regular physical activity contributes to improved life satisfaction. Therefore, it can be inferred that these emerging adult characteristics may positively influence life satisfaction through increased self-efficacy and engagement in physical activity.

By comparing the results of this study with existing literature, the importance of self-efficacy in the emerging adulthood period is further emphasized. The literature indicates that self-efficacy not only affects individual behavioral choices but is also closely related to mental health (24). The results of this study suggest that enhancing self-efficacy can serve as an effective strategy for improving life satisfaction, especially in contexts where emerging adults face identity and career uncertainties. Furthermore, the role of physical activity has been reinforced, supporting the view in health psychology that physical activity contributes not only to physical well-being but also to enhanced psychological well-being (32, 33).

In summary, this study demonstrates a significant association between the characteristics of emerging adulthood and life satisfaction, particularly through the mediating effects of self-efficacy and physical activity behavior. These findings are of considerable importance to educators and mental health professionals, as they can help better understand and address the challenges faced by emerging adults, thereby enhancing their life satisfaction. Given the notable connection between physical activity behavior and life satisfaction, health promotion activities should focus on enhancing the self-efficacy of emerging adults to encourage active participation in physical activities. Additionally, psychological interventions can provide necessary support and guidance for individuals experiencing challenges in identity exploration and construction during emerging adulthood. Educational institutions should also consider integrating topics related to self-efficacy and physical activity behavior into their curricula to promote student life satisfaction.

Overall, this study provides new insights into the factors affecting life satisfaction in emerging adults and points to practical applications that can initiate crucial improvements in life satisfaction for this group. Future research could further investigate other potential moderating variables, such as social support and environmental factors, to understand how they might influence these relationships, thereby offering more comprehensive support strategies for emerging adults.

### Limitations and advantages

This study has several limitations. Firstly, the sample selection primarily focused on Chinese university students, which may restrict the generalizability of the findings. Secondly, the measurement of life satisfaction was assessed through a single question, which may not comprehensively reflect all aspects of life satisfaction. Additionally, due to the non-normal distribution of the data, the Bollen–Stine bootstrap method was employed for statistical analysis. The Bollen–Stine bootstrap method is a non-parametric approach designed to estimate the distribution of statistics, particularly suitable for small samples and non-normal data. Although this method can provide more robust results, it may yield different outcomes compared to other statistical methods (such as traditional *t*-tests or ANOVA), necessitating caution in interpreting the results.

Despite these limitations, the study also presents several advantages. Firstly, it focuses on the life satisfaction of emerging adults, filling a gap in the existing literature. Secondly, the research considers multiple variables (such as self-efficacy and physical activity behavior) to analyze the factors influencing life satisfaction, thereby providing a more comprehensive perspective. Furthermore, the findings offer practical insights for educators and mental health professionals, assisting them in gaining a deeper understanding of the challenges faced by emerging adults.

Future research could explore several directions. Firstly, to validate the universality of the results, it is necessary to replicate these findings in a broader and more diverse population. Secondly, further exploration of the underlying mechanisms between the developmental features of emerging adulthood, self-efficacy, and physical activity behavior could be conducted. Additionally, to better understand causality, future studies may consider employing experimental designs or longitudinal research methods. Lastly, other variables that may be related to life satisfaction, such as social support, coping strategies, or life events, should also be investigated.

## Conclusion

This study validated our hypothesis model, which suggests that the developmental features of emerging adulthood may indirectly influence life satisfaction through their impact on self-efficacy and physical activity behavior. Specific intervention measures for educators and mental health professionals could include designing self-efficacy enhancement programs tailored specifically for emerging adults, such as workshops or group discussions aimed at boosting their self-efficacy. Additionally, university sports activity programs could be customized based on the research findings to encourage students to engage in more physical activities, thereby improving their life satisfaction. For example, schools could offer a variety of sports courses and activities to meet the diverse interests and needs of students, thereby promoting their participation in physical activities.

## Data Availability

The raw data supporting the conclusions of this article will be made available by the authors, without undue reservation.
